# Benefits and Safety of Astaxanthin in the Treatment of Mild-To-Moderate Dry Eye Disease

**DOI:** 10.3389/fnut.2021.796951

**Published:** 2022-01-13

**Authors:** Lei Tian, Ya Wen, Siyuan Li, Peng Zhang, Yinghui Wang, Jingyi Wang, Kai Cao, Lihua Du, Ningli Wang, Ying Jie

**Affiliations:** Beijing Ophthalmology and Visual Science Key Lab, Beijing Tongren Eye Center, Beijing Institute of Ophthalmology, Beijing Tongren Hospital, Capital Medical University, Beijing, China

**Keywords:** astaxanthin, dry eye disease, ocular surface, antioxidant, oxidative stress

## Abstract

**Objectives:** To evaluate the effect of astaxanthin in the treatment of mild-to-moderate dry eye disease (DED) in middle-aged and elderly patients.

**Methods:** 120 eyes of 60 middle-aged and elderly patients with mild-to-moderate DED were enrolled in this prospective, one-group, quasi-experimental study. Six milligram Astaxanthin tablets (Weihong Haematococcus Pluvialis Astaxanthin, Hangzhou Xinwei Low Carbon Technology R&D Co., Ltd., China) were administered orally, twice daily for 30 ± 2 days. History of eye diseases, treatment, systemic disease, and medication before the test were recorded. In addition, the ocular surface disease index (OSDI) questionnaire, non-invasive tear break-up time (NIBUT), fluorescein break-up time (FBUT), corneal fluorescein staining (CFS) score, eyelid margin signs, meibomian gland (MG) expressibility, meibum quality, meibomian gland dropout (MGDR), Schirmer I test (SIt), tear meniscus height (TMH), bulbar conjunctiva congestion degree, blink frequency, incomplete blink rate, and thickness of tear film lipid layer were collected before treatment, 2 weeks after the initiation of treatment, and at the end of treatment. Visual acuity (VA), intraocular pressure (IOP), anterior segment, fundus, discomfort symptoms and other adverse reactions were also monitored throughout the study to assess the safety.

**Results:** OSDI score, NIBUT, BUT, CFS score, eyelid margin signs, MG expressibility, meibum quality, and blink frequency improved significantly to varying degrees after treatment compared with those before the treatment (*P* < 0.05), while TMH, SIt, conjunctival congestion, the thickness of tear film lipid layer, MGDR, incomplete blink rate, VA and IOP did not differ (*P* > 0.05).

**Conclusions:** Oral administration of astaxanthin improves the symptoms and signs of middle-aged and elderly patients with mild-to-moderate DED.

## Introduction

According to the TFOS DEWS II published in 2017, dry eye disease (DED) is a multifactorial disease of the ocular surface characterized by the loss of homeostasis of the tear film and ocular symptoms, in which tear film instability and hyperosmolarity, ocular surface inflammation and damage, and neurosensory abnormalities play etiological roles (1). DED can cause a variety of ocular symptoms, such as dryness, burning, and blurred vision. If not treated properly, it causes ocular surface damage, leading to corneal complications and permanent vision loss (2). With the improvement in people's life and accelerated population aging, the prevalence of DED increases gradually. Currently, the global prevalence of DED ranges from 5 to 50% (3), and it is about 21–58% in China (4, 5). As a common ocular disease, DED affects patients' health and quality of life, and has even been associated with increased prevalence of depression (6).

DED is a disease caused by many factors such as environment, diet, trauma, drugs, local inflammation, living habits, and changes in body hormone levels (7). Among them meibomian gland dysfunction (MGD) is one of the most common causes of DED, resulting in lipid abnormalities of tear and evaporative DED (3). In middle-aged and older people (>40-years-old), the prevalence of MGD can reach 38–68% (3). This is probably due to the increase in oxidative stress with advanced age may lead to the loss of function of ocular surface glands, such as lacrimal glands and meibomian glands, resulting in reduced tear secretion and MGD (8, 9). Decreased tear secretion can lead to aqueous-deficient DED and MGD can lead to evaporative DED (1). The reduced secretion and increased evaporation cause hyperosmosis of tears, which increases the generation of reactive oxygen species (ROS) in corneal epithelial cells, disrupts the balance of oxygenase and antioxidants, and eventually induces oxidative stress (10). ROS cause membrane lipid peroxidation and mitochondrial DNA damage (11). These phenomena lead to mitochondrial dysfunction and increase cell damage in conjunctival epithelial tissue, lacrimal glands, meibomian gland, and other tissues secreting tears, thereby aggravating DED (12). Furthermore, oxidative stress can activate multiple inflammatory pathways and lead to ocular surface inflammation (10, 13). The concentration of inflammatory cytokines in tears of patients with DED is higher than that in healthy individuals and positively correlated with the severity of symptoms and signs of DED (14, 15). Oxidative stress and ocular surface inflammation complement each other in the pathogenesis of DED, suggesting that the oxidative stress pathway may be a new target for DED treatment. Presently, the mechanism of oxidative stress and related therapies of DED has gained increasing attention. Previous studies have shown that the human meibomian gland and conjunctival tissue contain a natural antioxidant system that resists the oxidation of secreted lipids and proteins (16). The imbalance of the local antioxidant system alters the lipid and mucin layers of the tear film, resulting in excessive evaporation and instability of the tear film and eventually DED (13). Some clinical studies have shown that the oral administration of compound preparations containing antioxidants, such as anthocyanin, astaxanthin, and vitamins A, C, and E, can reduce the concentration of ROS in tears, thereby increasing the production of tears and improving the stability of tear film (17, 18).

Astaxanthin is a naturally occurring carotenoid and the highest grade product of carotenoid synthesis that is recognized as one of the strongest antioxidants in nature (19). A series of *in vitro* and *in vivo* cell and animal experiments have demonstrated that astaxanthin increases local antioxidants, inactivates and scavenges oxygen free radicals, and inhibits the rise in age-related oxidative stress markers, such as p53, p21, and p16 (20, 21). Moreover, it downregulates the inflammatory factors, such as interleukin (IL)-1β, IL-6, and tumor necrosis factor-alpha (TNF-α) (22–24), and its antioxidant and anti-inflammatory effects are dose-dependent (25). These findings suggest that astaxanthin can reduce oxidative stress damage and inhibit the inflammatory response. In an *in vitro* study, astaxanthin was shown to inhibit the increase in the age-related oxidative stress markers in corneal epithelial cells (20) and the ocular surface inflammation by downregulating the expression of high mobility group box 1 protein (HMGB1) and inflammatory cytokines (TNF-α and IL-1β) (26), which has a potential therapeutic effect on DED. However, its clinical effect on DED is yet to be investigated. The present study aims to investigate the efficacy of astaxanthin in the treatment of mild-to-moderate DED through a single-group pretest-posttest study.

## Materials and Methods

### Study Design, Patients, and Ethics Approval

A prospective, single-center, one-group, pretest-posttest quasi-experimental study was conducted in 60 middle-aged and elderly patients with mild-to-moderate DED admitted to the Ophthalmology Department of Beijing Tongren Hospital, Capital Medical University from March 2021 to May 2021. A total of 120 eyes were assessed, including 18 eyes of 9 males and 102 eyes of 51 females with an average age of 60 (range: 51 to 72 years). The diagnosis of DED was based on the Diagnostic Methodology Report published by the Tear Film and Ocular Surface Society (TFOS) Dry Eye Workshop (DEWS) in 2017 (2). This study was reviewed and approved by the Ethics Committee of Beijing Tongren hospital and registered in the Chinese Clinical Trial Registry (Clinical Trial Registration No. ChiCTR2100044243). The patients and their families were informed of the examination content, treatment items, and matters needing attention before treatment, and all patients signed the informed consent.

### Inclusion Criteria

The patients in this study conformed to all the following inclusion criteria: (1) voluntarily participated in the clinical study and signed the informed consent; (2) no gender limitation, aged 50–80 years; (3) presence of clinical signs and symptoms of DED, and the clinical examination conformed to the diagnostic criteria for mild-to-moderate DED; (4) no staining or spotty staining of corneal fluorescein ≤ 30 points or no more than two quadrants; (5) tear film break-up time ≥ 2 s; (6) Schirmer I test (SIt) > 0 mm/5 min; (7) tear meniscus height (TMH) > 0 mm; (8) had not participated in clinical trials of other drugs within the past 2 weeks; and (9) had not taken any other medication for DED, or had stopped taking any other medication for > 2 weeks.

### Exclusion Criteria

Patients with a diagnosis not consistent with DED or any of the following conditions were excluded: (1) allergic to any ingredient in the test drug; (2) the patients were presumed to be at the active stage of fungal, bacterial, or viral keratitis and conjunctivitis; (3) the total corneal staining points were >30 points or more than two quadrants, with patchy staining fusion or filamentous material; (4) tear film break-up time < 2 s; (5) Schirmer I test (SIt) = 0 mm/5 min; (6) tear meniscus height (TMH) = 0 mm; (7) severe MGD patients (grade 4); (8) prolonged systemic or ocular medication may affect the study evaluation of patients; (9) Sjögren's syndrome, systemic diseases, and other ocular diseases that affect the evaluation of efficacy; and (10) any other condition that affecting the ocular surface and was not appropriate for inclusion in the study such as eye surgery, abnormal eyelid structure and so on.

### Treatment and Follow-Up

A total of 120 eyes of 60 middle-aged and elderly patients with mild-to-moderate DED who met the standards of enrollment were enrolled. The patients' history of eye diseases, treatment, systemic diseases, medication, subjective symptoms and objective signs before the test were recorded. Six milligram Astaxanthin tablets (6 mg astaxanthin/tablet, Weihong Haematococcus Pluvialis Astaxanthin, Hangzhou Xinwei Low Carbon Technology R&D Co., Ltd., China) were administered orally, twice daily for 30 ± 2 days. Then, the subjective symptoms and objective signs 2 weeks after the initiation and at the end of the treatment were recorded. All the objective checks were performed by the same experienced operator using the same equipment.

### Evaluation of Symptoms and Signs

#### Ocular Surface Disease Index (OSDI) Questionnaire

The OSDI questionnaire was used to evaluate the subjective ocular symptoms of patients in the past week (2). The questionnaire consisted of 12 questions, with a total score of 0–100, and assessed the severity of subjective symptoms of DED from three aspects: ocular symptoms, visual function, and environmental triggers. The higher the score, the more severe the dry eye symptoms.

#### Eyelid Margin Signs

The eyelid margin was observed and scored under the slit lamp based on the severity of signs. DED can cause inflammation of the eyelid margins (27) and the increase of inflammatory factor concentration in eyelid margin can lead to blood vessel dilation, new capillary formation, proliferation and keratosis of epithelial cells (28). This results in redness, thickening and blunt round eyelid margins. Moreover, meibum will also easily coagulate and be mixed with keratinized duct epithelial cells, blocking meibomian gland orifices (29). Eyelid margin signs were closely related to meibomian gland dropout (30), leading to MGD and eventually DED. The composite eyelid score was used to evaluate the eyelid margin signs (31), including blunt rounding shape of the eyelid margin, thickened lid margin, hyperkeratinization of the lid margin, congestion of the anterior lid margin, and vascularity and telangiectasia around meibomian gland orifices. Each clinical symptom was allocated a score of 1 point, on a scale of 0–5 points. The higher the score, the more severe the inflammation of eyelids and the more severe the MGD.

#### Meibomian Gland (MG) Expressibility

The central five glands of the lower eyelids were pressed with Meibomian Gland Evaluator (MGE; Tear Science, Inc., Milpitas, USA), and the amount of meibum extruded was observed to judge its expressibility (32). Meibum expressibility was thought to reflect meibomian gland function (2). The scoring criteria were as follows: 0, all glands expressible; 1, 3–4 glands expressible; 2, 1–2 glands expressible; 3, no glands expressible. The higher the score, the worse the MG expressibility.

#### Meibum Quality

Meibum was extracted from the central five meibomian glands by gently pressing 1–2 mm below the eyelid margin. The properties of meibum were observed and scored according to a previously proposed method (33). Meibum quality was also used to assess meibomian gland function (2). The scoring criteria were as follows: 0, clear fluid; 1, cloudy fluid; 2, cloudy particulate fluid; 3, inspissated, toothpaste-like discharge. The total score is between 0 and 15. The higher the score, the worse the quality of meibum.

#### Meibomian Gland Dropout (MGDR)

The meibomian gland was photographed by the Meibo-Scan of Oculus Keratograph 5M (K5M, Oculus Optikgerate GmbH, Germany). In infrared light, the meibomian gland showed white lines, while the other parts showed dark gray background. Blockage of the meibomian gland orifices leads to increased pressure in the meibomian gland, degenerative dilation, and eventual loss (30). The loss of the glands was divided into four grades (34): grade 0, no loss of meibomian glands; grade 1, loss of meibomian glands below 1/3rd of the total area; grade 2, loss of meibomian glands accounting for 1/3rd−2/3rd of the total area; and grade 3, loss of meibomian glands accounting for 2/3rd or more of the total area. The higher the score, the greater the meibomian gland loss area.

#### Tear Meniscus Height (TMH)

The white light tool of Oculus Keratograph 5M was used to measure the TMH image of the patient. TMH was formed by the tear gathered at the upper and lower eyelid margin, representing the amount of tear secretion and lacrimal gland function (35). The central TMH data of the lower eyelid was acquired by the measuring tool in the system and recorded in millimeters (mm). The higher the TMH value, the more tear secretion.

#### Schirmer I Test (SIt)

After the tears gathered at the lower eyelid margin were wiped, a 35 mm × 5 mm filter paper strip was placed in the conjunctival sac under the patient's eyes without topical anesthesia. SIt was mainly used to measure the basal and reflex secretions amount of the main and accessory lacrimal glands (36). The wetted length of the filter paper strip was observed after 5 min, and the tear secretion of the left and right eyes was recorded in mm. The longer the wetted paper, the more the tear secretion.

#### Corneal Fluorescein Staining (CFS)

One to two percentage fluorescein sodium dye was dropped into the conjunctival sac of the patient's lower eyelid. After 1 min, the cornea was observed under the cobalt blue light of a slit lamp microscope. The corneal epithelial defect site showed yellowish-green staining, and the fluorescein break-up time (FBUT) was recorded in second (s) for the unit. The corneal fluorescence staining was scored according to the American NEI scale (37). The cornea was divided into five regions and graded based on dye distribution. The CFS score was between 0 and 15, ranging from 0 to 3 in each region as follows: 0, no staining; 1, 1–30 punctate staining; 2, punctate staining >30; and 3, diffuse staining, filaments, and ulcer. The higher the score, the more severe the corneal epithelial defect. The longer the FBUT time, the more stable the tear film.

#### Noninvasive Tear Break-Up Time (NIBUT)

NIBUT was recorded using Oculus Keratograph 5M. After blinking twice, the patients were instructed to keep their eyes open. The system automatically recorded NIBUT and showed broken parts according to the inspection procedures for the formula to calculate the average NIBUT, with second (s) as the measuring unit (38). The longer the NIBUT time, the more stable the tear film.

#### Degree of Conjunctival Congestion

The conjunctival images of the patients were taken by the white light tool of Oculus Keratograph 5M, and the conjunctival congestion was evaluated and recorded by the image analysis technique. Inflammation is an important factor in the occurrence and development of DED, and conjunctival congestion is one of the most significant signs of ocular surface inflammation. The higher the grade, the more serious the congestion.

#### Lipid Layer Thickness (LLT) and Blink Analysis

The LipiView was used to measure LLT, blink frequency and incomplete blink proportion of eyes in 20 s. LLT reflects the ability of meibomian gland to secrete lipids (39). Blink abnormality is closely related to the occurrence and development of DED (40). The color unit of the interference image was converted to the thickness of the lipid layer of the tear film on the eye surface, the number of blinks in 20 s, and the proportion of incomplete blinks.

#### Safety Assessment

Visual acuity (VA) was measured using a standard logarithmic eye chart and intraocular pressure (IOP) was measured using a non-contact tonometer. Both eyes in all patients were monitored and recorded at the first and last follow-up. At the same time, the patients' anterior segment, fundus, discomfort symptoms and other adverse reactions were questioned and recorded.

### Statistical Analysis

SPSS 20.0 software was used for statistical analysis. In this study, the total OSDI score, NIBUT, FBUT, TMH, SIT, degree of conjunctival congestion, and LLT conformed to a normal distribution by W test, and the data are represented by mean ± standard deviation ($\bar{X}$ ± s). Before treatment, at 2 weeks after the initiation of treatment, and the end of treatment, no correlation was established between the OSDI score and the left and right eye data. One-way repeated-measures analysis of variance (ANOVA) was used for overall comparison, and Least Significant Difference (LSD)-*t*-test was used for pairwise comparison at different time. NIBUT, FBUT, TMH, SIt, degree of conjunctival congestion, LLT, and other data involving the correlation between the left and right eyes were collected bilaterally, and thus, a mixed-effect model was used for analysis. The corneal fluorescence staining score, eyelid margin changes, meibomian gland expressibility, meibum quality, meibomian gland dropout, blink frequency, incomplete blink proportion, VA and IOP did not conform to a normal distribution by W test and are represented by median (the first quartile value, the third quartile value) as M (Q1, Q3). Before treatment, 2 weeks after the initiation of treatment, and at the end of the treatment, the corneal fluorescence staining score, eyelid margin changes, meibomian gland expressibility, meibum quality, meibomian gland dropout, blink frequency, incomplete blink proportion, VA and IOP data were collected bilaterally, which were analyzed by mixed-effects model. *P* < 0.05 indicated statistical significance.

## Results

### Changes in OSDI Score, NIBUT, FBUT, TMH, SIt, LLT, and Severity of Conjunctival Congestion Before and After Treatment

The overall OSDI scores before and after treatment showed a statistically significant difference (Table 1). Moreover, a statistically significant difference was detected in the overall NIBUT, FBUT of patients before and after treatment (Table 2). Before and after treatment, no statistically significant difference was observed in the TMH, SIt, conjunctival congestion degree, and LLT of the tear film (all *P* > 0.05) (Table 2).

**Table 1:** Changes in OSDI score before and after treatment.

**Time**	**Number**	**OSDI score**
Before	60	52 ± 19
14 days later	60	42 ± 15^a^
30 days later	60	30 ± 16^a^^b^
*P*-value		<0.001

a *P < 0.05 vs. before treatment*;

b *P < 0.05 vs. 14 days after treatment*.

*Data are mean and standard deviation (SD)*.

*OSDI, ocular surface disease index*.

**Table 2:** Changes in NIBUT, FBUT, TMH, SIt, LLT, and conjunctival congestion degree before and after treatment.

**Time**	**Number**	**NIBUT (s)**	**FBUT (s)**	**TMH (mm)**	**SIt (mm)**	**Conjunctival congestion degree**	**LLT (nm)**
Before	120	7.18 ± 3.28	3.94 ± 1.58	0.23 ± 0.11	7.74 ± 6.33	1.34 ± 0.44	71.90 ± 23.77
14 days later	120	8.31 ± 3.91^a^	4.53 ± 2.29^a^	0.23 ± 0.09	7.70 ± 6.42	1.42 ± 0.45	74.55 ± 25.27
30 days later	120	8.34 ± 3.44^a^	4.69 ± 2.12^a^	0.23 ± 0.07	6.98 ± 6.61	1.39 ± 0.46	75.00 ± 24.03
*P*-value		0.016	0.011	0.880	0.458	0.397	0.567

a *P < 0.05 vs. before treatment*.

*Data are mean and standard deviation (SD)*.

*NIBUT, noninvasive tear break-up time; FBUT, fluorescein break-up time; TMH, tear meniscus height; SIt, Schirmer I test; LLT, lipid layer thickness*.

### Changes in CFS Score, Eyelid Margin Changes, MG Expressibility, Meibum Quality, MGDR, Blink Frequency, and Incomplete Blink Proportion Before and After Treatment

The overall comparison of CFS score, eyelid margin signs, MG expressibility, meibum quality, blink frequency before and after treatment showed a statistically significant difference (Table 3). The mean blink frequency was 24.9, 21.3, and 20.4 per minute before, 14 and 30 days after the initiation of treatment, respectively. Before and after treatment, no statistical significance was detected in the MGDR and incomplete blink proportion (all *P* > 0.05) (Table 3).

**Table 3:** Changes in CFS score, eyelid margin changes, MG expressibility, meibum quality, MGDR, blink frequency, and incomplete blink proportion before and after treatment.

**Time**	**Number**	**CFS score**	**Eyelid margin signs**	**MG expressibility**	**Meibum quality**	**MGDR (upper eyelid)**	**MGDR (lower eyelid)**	**Blink frequency (per minute)**	**Incomplete blink proportion**
Before	120	1 (0, 1)	1 (1, 1)	1 (1, 2)	1 (1, 1)	1 (1, 2)	1 (1, 2)	27 (12, 33)	0.75 (0.22, 1.00)
14d later	120	1 (0, 1)	1 (1, 1)	1 (1, 2)	1 (0, 1)^a^	1 (1, 2)	1 (1, 2)	18 (12, 27)^a^	0.88 (0.50, 1.00)
30 d later	120	0 (0, 2)^a^^b^	1 (0, 1)^a^^b^	1 (1, 1)^a^^b^	1 (0, 1)^a^	1 (1, 2)	1 (1, 1)	18 (9, 27)^a^	0.88 (0.50, 1.00)
*P*-value		<0.001	<0.001	<0.001	<0.001	0.183	0.407	0.020	0.086

a *P < 0.05 vs. before treatment*;

b *P < 0.05 vs. 14 days after treatment*.

*Data are median, the first quartile and the third quartile*.

*CFS, corneal fluorescein staining; MG, meibomian gland; MGDR, meibomian gland dropout*.

### Changes in VA, IOP, and Other Safety Assessment Before and After Treatment

There were no significant changes in visual acuity and intraocular pressure before and after treatment (all *P* > 0.05) (Table 4). The anterior segment and fundus in all patients also had no significant changes. None of the patients complained of other adverse reactions.

**Table 4:** Changes in VA and IOP before and after treatment.

**Time**	**Number**	**VA**	**IOP (mmHg)**
Before	120	0.3 (0.6, 0.8)	12.5 (10.8, 14.9)
30 days later	120	0.3 (0.7, 0.9)	13.0 (11.8, 14.8)
*P*-value		0.812	0.387

*Data are median, the first quartile and the third quartile*.

*VA, visual acuity; IOP, intraocular pressure*.

## Discussion

With the deepening of the understanding of the etiology and pathogenesis of DED, the promotion of inflammation and oxidative stress on the occurrence and development of DED and their interaction has been under intensive focus (13, 41, 42). Currently, anti-inflammatory drugs, including glucocorticoid, cyclosporine A, and tacrolimus, have been marketed for the treatment of DED (43). Only a few clinical studies have addressed the application of antioxidants in DED. A study found that oxidative stress promotes the production of reactive oxygen molecules, induces ocular surface epithelial damage, and promotes the occurrence and development of DED (44). Therefore, it is crucial to explore the effects of oxidative stress and antioxidant therapy on DED to elucidate the pathogenesis of DED and explore novel therapeutic targets. Astaxanthin has good antioxidant properties and can be industrially produced. Presently, the main industrial production method of astaxanthin is through the culture of *Rhodococcus* (45). Some studies have shown that the accumulation of astaxanthin in the ocular tissues of rats fed with astaxanthin-rich *Rhodococcus* can reach the maximum level within 6 h (46), indicating good bioavailability and the potential of achieving an effective blood concentration and playing a role in resisting oxidative stress in the eye. Therefore, astaxanthin from *Rhodiococcus* was selected to explore its therapeutic effect on mild to moderate middle-aged and elderly patients with DED.

This prospective, single-center, one-group pretest-posttest quasi-experimental study was conducted to observe the improvement in subjective symptoms and objective signs of mild-to-moderate DED in middle-aged and elderly patients with different etiologies after a daily supplement of 12 mg astaxanthin for 14 and 30 days. The results showed that OSDI score, CFS score, NIBUT, FBUT, meibum quality, and blink frequency were significantly improved on day 14 after daily astaxanthin supplementation compared with that before treatment. Both OSDI score and CFS score showed a tendency to improve with the duration of treatment. NIBUT, FBUT, and blink frequency were significantly improved 14 days after the initiation of the treatment. Although MG expressibility and eyelid margin signs were not significantly improved on day 14, they were significantly improved on day 30 compared with that before administration. Also, no significant differences were detected in the MGDR, TMH, SIt, LLT, conjunctival congestion degree, and incomplete blink.

OSDI is the score representing patients' subjective symptoms related to the severity of DED signs. In the current study, the patients' OSDI scores continued to decline, which might be attributable to astaxanthin blocking oxidative stress injury by increasing the level of antioxidant substances in the eye and inactivating and scavenging oxygen free radicals while downregulating inflammatory factors, such as interleukin (IL)-1β, IL-6, and tumor necrosis factor (TNF-α) (26). Therefore, astaxanthin may alleviate local inflammatory response, reduce the severity of DED signs and relieve the symptoms.

Corneal fluorescence staining score reflects the degree of the corneal epithelial defect and is an objective index to measure the severity of DED. The results of this study showed that after oral astaxanthin tablets, the CFS score of patients showed a downward trend compared with that before the treatment, and the corneal epithelial defect was improved continuously, which was greater at 30 days than at 14 days after oral administration. Some animal experiments have shown that astaxanthin improved the corneal fluorescence staining and reduced the expression of age-related markers in the dry eyes of rats (47). Thus, this phenomenon may be related to the fact that astaxanthin is an effective antioxidant, reduces local oxidative damage and inflammatory response, and provides a local microenvironment for the repair of corneal epithelial cells. It usually takes a while before the local microenvironment becomes suitable for corneal epithelial proliferation and hence, the speed of corneal epithelial repair is slow in the initial 14 days. Well-controlled inflammation and a local microenvironment suitable for cell proliferation could explain the marked improvement of corneal epithelial defect at day 30.

NIBUT and FBUT mainly reflect the stability of the tear film. The tear film stability is related to several factors, such as the changes in tear composition, tear secretion, and mucin secretion (7). In the current study, NIBUT and FBUT showed an overall upward trend, and the increase was obvious in the first 14 days, which might be related to the improvement in the secretion properties of the ocular glands. Since the control of inflammation in the first 14 days improved the structure and composition and greatly increased the stability of the tear film, the NIBUT and FBUT were prolonged. The level of oxidative stress in conjunctival goblet cells in middle-aged and elderly individuals with DED is high (48). The antioxidant and anti-inflammatory effects of astaxanthin may improve the inflammatory response of conjunctival goblet cells, thereby promoting mucin secretion, playing the role of mucin anchor, and increasing tear film stability.

The eyelid margin signs, MG expressibility, meibum quality, and MGDR could reflect the degree of eyelid margin inflammation and the function of the meibomian gland. Compared with before treatment, all three parameters were improved continuously, and the improvement in meibum quality was significant at 14 days after the initiation of treatment, while the changes in eyelid margin signs and the enhanced MG expressibility were more obvious at 30 days. The meibomian gland of DED patients is in a state of high oxidative stress (16, 41). Astaxanthin could improve the surrounding environment of meibomian gland cells, reduce oxidative stress and inflammation, which would help improve the amount and quality of meibum secretion. However, the increase of MG expressibility was a slow process, and hence, its improvement lagged slightly behind that of meibum quality. Astaxanthin improved the oxidative stress and inflammation of the eyelids, which helped reduce inflammation edema, inhibit angiogenesis, vasodilatation and keratosis. Therefore, the eyelid margin signs score decreased. However, the improvement of eyelid margin was not obvious in the first 14 days, which was believed to be the reason that keratosis of the eyelid margin and congested vessels needed a period of time to be absorbed. No significant difference was detected in MGDR before and after treatment, which was related to the irreversibility.

LLT is affected by the amount and quality of meibum secreted by the meibomian glands (49). The lipid layer of the tear film is composed of meibum secreted by the meibomian glands. The decrease in meibum secretion can directly lead to the decrease of LLT (50). Reduced meibum quality can increase tear film instability, resulting in uneven distribution of tear film on the ocular surface, and thereby affecting the LLT value. LLT increased after the treatment, albeit not significantly. Moreover, this phenomenon could also be accounted for a low baseline level of tear film lipid layer thickness in patients with mild-to-moderate DED. The improvement in the secretion capacity of the meibomian gland was not obvious in the first 14 days, and it would result in less increase in the thickness of the lipid layer of the tear film.

Change in the blink characteristics is a major contributing factor to DED, mainly including blink frequency and incomplete blink proportion. These two characteristics are affected by many factors, such as mental state, attention, physical activity, eye contact, and environment (40). Under normal circumstances, the average number of blinks per minute is 15–20 (7), i.e., the number of blinks per 20 s is about 5–7. In the current study, the number of blinks in 20 s was measured. According to the results, the blink frequency decreased gradually. Pretreatment blink frequency was higher than the normal value, which might be related to ocular surface symptoms and signs of patients with DED. Patients with poor tear film stability, shortened BUT, and severe DED symptoms, could only rely on increased blink frequency to achieve an optimal visual effect. However, after treatment, the blink frequency decreased gradually, albeit in some individuals, it was still higher than normal levels. Combined with the BUT, the time also increased but was still shorter than normal, which could be attributed to the blink frequency limited by the short BUT. In addition, the patients' OSDI score decreased but was higher than normal, indicating that the patients' symptoms had not disappeared completely, and long-term efficacy of astaxanthin is yet to be observed. Incomplete blinks are usually associated with long-term use of video terminals or other factors that impede eyelid closure (51, 52). Incomplete blink proportion had no significant change before and after treatment. This indicated that astaxanthin could not affect the method of blinking.

Conjunctival congestion degree is a major indicator of ocular surface inflammation. In this study, the degree of conjunctival congestion in patients was not significantly improved compared with that before the treatment, which might be due to the fact that the local anti-inflammatory effect of astaxanthin takes a long time to effectuate, the degree of ocular surface inflammation in patients with mild-to-moderate DED was mild or the short test time and small sample size.

The TMH and SIt reflected the lacrimal gland secretion capacity. In this study, TMH and SIt of patients before and after treatment did not improve significantly, which is similar to the results of previous animal experiments (47). This phenomenon could be attributed to the lack of a promoting effect on lacrimal gland secretion or failure to achieve an effective concentration of astaxanthin in the local lacrimal gland, thereby resulting in an inadequate improvement of lacrimal eye inflammation. It may also be related to the short test time and small sample size.

There were no significant changes in visual acuity, intraocular pressure, anterior segment and fundus before and after treatment. During the experiment, the subjects showed adaptability and no adverse reactions or complications. Given astaxanthin is administered orally, there is no ocular irritation. These results indicated that astaxanthin tablets could exert antioxidant, anti-inflammatory, ocular surface repair functions without causing discomfort. Additionally, the patients showed high medication compliance. Therefore, oral astaxanthin could be considered a safe method for the treatment of DED.

Nevertheless, the present study has some shortcomings. First, this study was a single-group design and had no control group. Although the changes in patients' conditions before and after astaxanthin intervention could be obtained, the placebo effect was ignored. Second, the observation time of this study was only about 1 month, and hence, it is impossible to predict the long-term efficacy of astaxanthin on DED. Third, the tear composition of the patients was not tested and systemic and local oxidative stress was not measured, rendering it impossible to observe whether astaxanthin reduces the oxidation in the tear. However, a range of measures were taken to improve the credibility of our study. The authors ensured that all patients were examined by the same experienced ophthalmologist, explained the questions before completing the OSDI questionnaire and allowed adequate time to answer it. Herein, the authors would propose a follow-up study to set up a reasonable control group, prolonged observation time for the observation of long-term curative effect and adverse reactions, in which case the sample size would be increased and the tear composition detection would be added, so that the efficacy of the astaxanthin in patients of different gender and age groups could be observed.

## Conclusions

In conclusion, as a comfortable option with fewer adverse reactions, oral administration of astaxanthin could serve as an effective treatment of DED by improving the tear film stability, the repair of corneal and conjunctival epithelial cells and the secretion function of the meibomian gland, improving subjective symptoms of DED. However, its effect on promoting lacrimal gland secretion was observed to be limited. The single-group design and lack of molecular biological detection constitute limitations of the current study, thus further studies with more robust methodology, such as randomized controlled trials and tests for inflammatory factors and oxidative stress are needed. Astaxanthin is of the potential to be a new choice for the treatment of mild to moderate DED.

## Data Availability Statement

The raw data supporting the conclusions of this article will be made available by the authors, without undue reservation.

## Ethics Statement

The studies involving human participants were reviewed and approved by Ethics Committee of Beijing Tongren hospital. The patients/participants provided their written informed consent to participate in this study.

## Author Contributions

LT contributed to the conception of the study. LT and YWe prepared the first draft. YWe, SL, PZ, YWa, and JW performed the experiment and recorded the data. KC and LD contributed significantly to the data analysis. NW and YJ provided constructive suggestions regarding the manuscript preparation, the data analysis and the interpretation of the results, and made critical revisions to the manuscript. All authors contributed to the article and approved the submitted version.

## Funding

This research was supported by the Open Research Fund from Beijing Advanced Innovation Center for Big Data-Based Precision Medicine, Beijing Tongren Hospital, Beihang University & Capital Medical University (BHTR-KFJJ-202001).

## Conflict of Interest

The authors declare that the research was conducted in the absence of any commercial or financial relationships that could be construed as a potential conflict of interest.

## Publisher's Note

All claims expressed in this article are solely those of the authors and do not necessarily represent those of their affiliated organizations, or those of the publisher, the editors and the reviewers. Any product that may be evaluated in this article, or claim that may be made by its manufacturer, is not guaranteed or endorsed by the publisher.

## References

[1] Craig JP Nichols KK Akpek EK Caffery B Dua HS Joo CK. TFOS DEWS II definition and classification report. Ocul Surf. (2017) 15:276–83. 10.1016/j.jtos.2017.05.008 28736335

[2] Wolffsohn JS Arita R Chalmers R Djalilian A Dogru M Dumbleton K. TFOS DEWS II diagnostic methodology report. Ocul Surf. (2017) 15:539–74. 10.1016/j.jtos.2017.05.001 28736342

[3] Stapleton F Alves M Bunya VY Jalbert I Lekhanont K Malet F. TFOS DEWS II epidemiology report. Ocul Surf. (2017) 15:334–65. 10.1016/j.jtos.2017.05.003 28736337

[4] Song P Xia W Wang M Chang X Wang J Jin S. Variations of dry eye disease prevalence by age, sex and geographic characteristics in China: a systematic review and meta-analysis. J Glob Health. (2018) 8:020503. 10.7189/jogh.08.020503 30206477 PMC6122008

[5] Zhang S Hong J. Risk factors for dry eye in mainland China: a multi-center cross-sectional hospital-based study. Ophthalmic Epidemiol. (2019) 26:393–9. 10.1080/09286586.2019.1632905 31218906

[6] Zheng Y Wu X Lin X Lin H. The prevalence of depression and depressive symptoms among eye disease patients: a systematic review and meta-analysis. Sci Rep. (2017) 7:46453. 10.1038/srep46453 28401923 PMC5388862

[7] Bron AJ de Paiva CS Chauhan SK Bonini S Gabison EE Jain S. TFOS DEWS II pathophysiology report. Ocul Surf. (2017) 15:438–510. 10.1016/j.jtos.2017.05.011 28736340

[8] Rocha EM Alves M Rios JD Dartt DA. The aging lacrimal gland: changes in structure and function. Ocul Surf. (2008) 6:162–74. 10.1016/S1542-0124(12)70177-5 18827949 PMC4205956

[9] Borchelt DR Ibrahim OMA Dogru M Matsumoto Y Igarashi A Kojima T. Oxidative stress induced age dependent meibomian gland dysfunction in Cu, Zn-superoxide dismutase-1 (Sod1) knockout mice. PLoS ONE. (2014) 9:e99328. 10.1371/journal.pone.0099328 25036096 PMC4103776

[10] Chen Y Li M Li B Wang W Lin A Sheng M. Effect of reactive oxygen species generation in rabbit corneal epithelial cells on inflammatory and apoptotic signaling pathways in the presence of high osmotic pressure. PLoS ONE. (2013) 8:e72900. 10.1371/journal.pone.0072900 23977369 PMC3744495

[11] Slimen IB Najar T Ghram A Dabbebi H Ben Mrad M Abdrabbah M. Reactive oxygen species, heat stress and oxidative-induced mitochondrial damage. A review. Int J Hyperthermia. (2014) 30:513–23. 10.3109/02656736.2014.971446 25354680

[12] Deng R Hua X Li J Chi W Zhang Z Lu F. Oxidative stress markers induced by hyperosmolarity in primary human corneal epithelial cells. PLoS ONE. (2015) 10:e0126561. 10.1371/journal.pone.0126561 26024535 PMC4449087

[13] Dogru M Kojima T Simsek C Tsubota K. Potential role of oxidative stress in ocular surface inflammation and dry eye disease. Invest Ophthalmol Vis Sci. (2018) 59:DES163–8. 10.1167/iovs.17-23402 30481822

[14] Lee SY Han SJ Nam SM Yoon SC Ahn JM Kim TI. Analysis of tear cytokines and clinical correlations in Sjögren syndrome dry eye patients and non-Sjögren syndrome dry eye patients. Am J Ophthalmol. (2013) 156:247–53.e1. 10.1016/j.ajo.2013.04.003 23752063

[15] Aragona P Aguennouz M Rania L Postorino E Sommario MS Roszkowska AM. Matrix metalloproteinase 9 and transglutaminase 2 expression at the ocular surface in patients with different forms of dry eye disease. Ophthalmology. (2015) 122:62–71. 10.1016/j.ophtha.2014.07.048 25240629

[16] Nezzar H Mbekeani JN Noblanc A Chiambaretta F Drevet JR Kocer A. Investigation of antioxidant systems in human meibomian gland and conjunctival tissues. Exp Eye Res. (2017) 165:99–104. 10.1016/j.exer.2017.09.005 28958587

[17] Huang JY Yeh PT Hou YC. A randomized, double-blind, placebo-controlled study of oral antioxidant supplement therapy in patients with dry eye syndrome. Clin Ophthalmol. (2016) 10:813–20. 10.2147/OPTH.S106455 27274185 PMC4869783

[18] Kojima T Dogru M Kawashima M Nakamura S Tsubota K. Advances in the diagnosis and treatment of dry eye. Prog Retinal Eye Res. (2020) 78:100842. 10.1016/j.preteyeres.2020.100842 32004729

[19] Higuera-Ciapara I Félix-Valenzuela L Goycoolea FM. Astaxanthin: a review of its chemistry and applications. Crit Rev Food Sci Nutr. (2006) 46:185–96. 10.1080/10408690590957188 16431409

[20] Shimokawa T Yoshida M Fukuta T Tanaka T Inagi T Kogure K. Efficacy of high-affinity liposomal astaxanthin on up-regulation of age-related markers induced by oxidative stress in human corneal epithelial cells. J Clin Biochem Nutr. (2019) 64:27–35. 10.3164/jcbn.18-27 30705509 PMC6348414

[21] Sudharshan SJ Dyavaiah M. Astaxanthin protects oxidative stress mediated DNA damage and enhances longevity in Saccharomyces cerevisiae. Biogerontology. (2021) 22:81–100. 10.1007/s10522-020-09904-9 33108581

[22] Fang Q Guo S Zhou H Han R Wu P Han C. Astaxanthin protects against early burn-wound progression in rats by attenuating oxidative stress-induced inflammation and mitochondria-related apoptosis. Sci Rep. (2017) 7:41440. 10.1038/srep41440 28128352 PMC5269753

[23] Yeh PT Huang HW Yang CM Yang WS Yang CH. Astaxanthin inhibits expression of retinal oxidative stress and inflammatory mediators in streptozotocin-induced diabetic rats. PLoS ONE. (2016) 11:e0146438. 10.1371/journal.pone.0146438 26765843 PMC4713224

[24] Chang MX Xiong F. Astaxanthin and its effects in inflammatory responses and inflammation-associated diseases: recent advances and future directions. Molecules. (2020) 25:5342. 10.3390/molecules25225342 33207669 PMC7696511

[25] Otsuka T Shimazawa M Inoue Y Nakano Y Ojino K Izawa H. Astaxanthin protects against retinal damage: evidence from *in vivo* and *in vitro* retinal ischemia and reperfusion models. Curr Eye Res. (2016) 41:1465–72. 10.3109/02713683.2015.1127392 27158842

[26] Li H Li J Hou C Li J Peng H Wang Q. The effect of astaxanthin on inflammation in hyperosmolarity of experimental dry eye model *in vitro* and *in vivo*. Exp Eye Res. (2020) 197:108113. 10.1016/j.exer.2020.108113 32531188

[27] Rynerson JM Perry HD. DEBS - a unification theory for dry eye and blepharitis. Clin Ophthalmol. (2016) 10:2455–67. 10.2147/OPTH.S114674 28003734 PMC5158179

[28] Amescua G Akpek EK Farid M Garcia-Ferrer FJ Lin A Rhee MK. American academy of ophthalmology preferred practice pattern, and p. External disease, blepharitis preferred practice pattern(R). Ophthalmology. (2019) 126:P56–93. 10.1016/j.ophtha.2018.10.019 30366800

[29] Bernardes TF Bonfioli AA. Blepharitis. Semin Ophthalmol. (2010) 25:79–83. 10.3109/08820538.2010.488562 20590417

[30] Ha M Kim JS Hong SY Chang DJ Whang WJ Na KS. Relationship between eyelid margin irregularity and meibomian gland dropout. Ocul Surf. (2021) 19:31–7. 10.1016/j.jtos.2020.11.007 33246034

[31] Yan X Hong J Jin X Chen W Rong B Feng Y. The efficacy of intense pulsed light combined with meibomian gland expression for the treatment of dry eye disease due to meibomian gland dysfunction: a multicenter, randomized controlled trial. Eye Contact Lens. (2021) 47:45–53. 10.1097/ICL.0000000000000711 32452923 PMC7752242

[32] Pflugfelder SC Tseng SC Sanabria O Kell H Garcia CG Felix C. Evaluation of subjective assessments and objective diagnostic tests for diagnosing tear-film disorders known to cause ocular irritation. Cornea. (1998) 17:38–56. 10.1097/00003226-199801000-00007 9436879

[33] Bron AJ Benjamin L Snibson GR. Meibomian gland disease. Classification and grading of lid changes. Eye. (1991) 5(Pt. 4):395–411. 10.1038/eye.1991.65 1743355

[34] Srinivasan S Menzies K Sorbara L Jones L. Infrared imaging of meibomian gland structure using a novel keratograph. Optom Vis Sci. (2012) 89:788–94. 10.1097/OPX.0b013e318253de93 22525129

[35] Tung CI Perin AF Gumus K Pflugfelder SC. Tear meniscus dimensions in tear dysfunction and their correlation with clinical parameters. Am J Ophthalmol. (2014) 157:301–10 e1. 10.1016/j.ajo.2013.09.024 24315297 PMC3946984

[36] Brott NR Ronquillo Y. Schirmer Test. Treasure Island, FL: StatPearls Publishing Copyright © 2021, StatPearls Publishing LLC (2021). 32644585

[37] Amparo F Wang H Yin J Marmalidou A Dana R. Evaluating corneal fluorescein staining using a novel automated method. Invest Ophthalmol Vis Sci. (2017) 58:BIO168–73. 10.1167/iovs.17-21831 28693042

[38] Lee R Yeo S Aung HT Tong L. Agreement of noninvasive tear break-up time measurement between Tomey RT-7000 auto refractor-keratometer and oculus keratograph 5M. Clin Ophthalmol. (2016) 10:1785–90. 10.2147/OPTH.S110180 27695283 PMC5033501

[39] Li J Ma J Hu M Yu J Zhao Y. Assessment of tear film lipid layer thickness in patients with Meibomian gland dysfunction at different ages. BMC Ophthalmol. (2020) 20:394. 10.1186/s12886-020-01667-8 33023522 PMC7539430

[40] Tsubota K Hata S Okusawa Y Egami F Ohtsuki T Nakamori K. Quantitative videographic analysis of blinking in normal subjects and patients with dry eye. Arch Ophthalmol. (1996) 114:715–20. 10.1001/archopht.1996.01100130707012 8639084

[41] Seen S Tong L. Dry eye disease and oxidative stress. Acta Ophthalmol. (2018) 96:e412–20. 10.1111/aos.13526 28834388

[42] Yamaguchi T. Inflammatory response in dry eye. Invest Ophthalmol Vis Sci. (2018) 59:Des192–9. 10.1167/iovs.17-23651 30481826

[43] Jones L Downie LE Korb D Benitez-Del-Castillo JM Dana R Deng SX. TFOS DEWS II management and therapy report. Ocul Surf. (2017) 15:575–628. 10.1016/j.jtos.2017.05.006 28736343

[44] Uchino Y Kawakita T Ishii T Ishii N Tsubota K. A new mouse model of dry eye disease: oxidative stress affects functional decline in the lacrimal gland. Cornea. (2012) 31(Suppl. 1):S63–7. 10.1097/ICO.0b013e31826a5de1 23038038

[45] Ambati RR Phang SM Ravi S Aswathanarayana RG. Astaxanthin: sources, extraction, stability, biological activities and its commercial applications–a review. Mar Drugs. (2014) 12:128–52. 10.3390/md12010128 24402174 PMC3917265

[46] Ranga Rao Raghunath Reddy RL Baskaran V Sarada R Ravishankar GA. Characterization of microalgal carotenoids by mass spectrometry and their bioavailability and antioxidant properties elucidated in rat model. J Agric Food Chem. (2010) 58:8553–9. 10.1021/jf101187k 20681642

[47] Shimokawa T Fukuta T Inagi T Kogure K. Protective effect of high-affinity liposomes encapsulating astaxanthin against corneal disorder in the *in vivo* rat dry eye disease model. J Clin Biochem Nutr. (2020) 66:224–32. 10.3164/jcbn.19-102 32523249 PMC7263926

[48] de Souza RG Yu Z Hernandez H Trujillo-Vargas CM Lee A Mauk KE. Modulation of oxidative stress and inflammation in the aged lacrimal gland. Am J Pathol. (2021) 191:294–308. 10.1016/j.ajpath.2020.10.013 33159886 PMC7888192

[49] Finis D Pischel N Borrelli M Schrader S Geerling G. Einflussfaktoren auf die messung der lipidschichtdicke des tränenfilms mittels interferometrie. Klin Monatsblätter Augenheilkunde. (2014) 231:603–10. 10.1055/s-0034-1368536 24940758

[50] Chou YB Fan NW Lin PY. Value of lipid layer thickness and blinking pattern in approaching patients with dry eye symptoms. Can J Ophthalmol. (2019) 54:735–40. 10.1016/j.jcjo.2019.03.005 31836108

[51] Talens-Estarelles C Garcia-Marques JV Cervino A Garcia-Lazaro S. Use of digital displays and ocular surface alterations: a review. Ocul Surf. (2021) 19:252–65. 10.1016/j.jtos.2020.10.001 33053438

[52] Park J Baek S. Dry eye syndrome in thyroid eye disease patients: the role of increased incomplete blinking and Meibomian gland loss. Acta Ophthalmol. (2019) 97:e800–6. 10.1111/aos.14000 30593716

